# OxyR-regulated T6SS functions in coordination with siderophore to resist oxidative stress

**DOI:** 10.1128/spectrum.03231-23

**Published:** 2024-01-08

**Authors:** Changfu Li, Zhiyan Wei, Xinquan He, Haiyang He, Yuqi Liu, Yuxin Zuo, He Xiao, Yao Wang, Xihui Shen, Lingfang Zhu

**Affiliations:** 1 State Key Laboratory of Crop Stress Biology for Arid Areas, Shaanxi Key Laboratory of Agricultural and Environmental Microbiology, College of Life Sciences, Northwest A&F University, Yangling, Shaanxi, China; Institut Pasteur, Paris, France

**Keywords:** OxyR, type VI secretion system, siderophore, iron, oxidative stress

## Abstract

The formation of reactive oxygen species is harmful and can destroy intracellular macromolecules such as lipids, proteins, and DNA, even leading to bacterial death. To cope with this situation, microbes have evolved a variety of sophisticated mechanisms, including antioxidant enzymes, siderophores, and the type VI secretion system (T6SS). However, the mechanism of oxidative stress resistance in *Cupriavidus pinatubonensis* is unclear. In this study, we identified Reut_A2805 as an OxyR ortholog in *C*. *pinatubonensis*, which positively regulated the expression of T6SS1 by directly binding to its operon promoter region. The study revealed that OxyR-regulated T6SS1 combats oxidative stress by importing iron into bacterial cells. Moreover, the T6SS1-mediated outer membrane vesicles-dependent iron acquisition pathway played a crucial role in the oxidative stress resistance process. Finally, our study demonstrated that the T6SS1 and siderophore systems in *C. pinatubonensis* exhibit different responses in combating oxidative stress under low-iron conditions, providing a comprehensive understanding of how bacterial iron acquisition systems function in diverse conditions.

**IMPORTANCE:**

The ability to eliminate reactive oxygen species is crucial for bacterial survival. Continuous formation of hydroperoxides can damage metalloenzymes, disrupt DNA integrity, and even result in cell death. While various mechanisms have been identified in other bacterial species to combat oxidative stress, the specific mechanism of oxidative stress resistance in *C. pinatubonensis* remains unclear. The importance of this study is that we elucidate the mechanism that OxyR-regulated T6SS1 combats oxidative stress by importing iron with the help of bacterial outer membrane vesicle. Moreover, the study highlights the contrasting responses of T6SS1- and siderophore-mediated iron acquisition systems to oxidative stress. This study provides a comprehensive understanding of bacterial iron acquisition and its role in oxidative stress resistance in *C. pinatubonensis* under low-iron conditions.

## INTRODUCTION

Oxic environments are hostile to most organisms because oxygen is a reactive chemical, which continuously forms intracellular superoxide anions (O_2_^•−^), hydrogen peroxide (H_2_O_2_), and hydroxyl radicals (OH^•^) (1, 2). Such reactive oxygen species (ROS) can destroy the structure and activity of metalloenzymes and the integrity of DNA, resulting in bacterial death or bacteriostasis (3). To survive those adverse conditions, bacteria have developed elegant adaptive strategies to defend themselves. Bacteria sense and respond to oxidative stress using transcription factors, such as OxyR and SoxRS (4, 5). These transcription factors coordinate specific ROS stress tolerance mechanisms, including the synthesis of antioxidant enzymes and small-molecular-weight antioxidants, which eliminate the adverse effects of ROS and maintain redox homeostasis (3, 6, 7). For example, OxyR in many bacteria facilitates infection by inducing catalase production to degrade hydrogen peroxide generated by the host defense response (5, 8, 9). Zinc (Zn^2+^) and manganese (Mn^2+^) play important roles in oxidative stress protection by functioning as cofactors or structural components of antioxidant enzymes, as well as forming low-molecular-weight antioxidant complexes (10, 11). These metal ion transporters, such as MntH and ZinT/ZnuABC, are typically under the control of oxidative stress transcriptional regulators (4).

The type VI secretion system (T6SS) is a widely distributed transmembrane nanomachine that resembles inverted contractile bacteriophage tails, which functions to inject effector proteins extracellularly or into neighboring cells (9, 12–16). Initially, the T6SS was thought to transport effector proteins with activities against prokaryotic and eukaryotic cells, thereby providing bacteria with survival advantages in microbe-microbe interactions and microbe-host interactions (17–20). T6SS is also involved in the oxidative stress response in bacterial pathogens, thereby improving their survival (9, 21–23). In addition to playing a vital role in metal ion uptake to support survival in metal-restricted environments, T6SS is implicated in resistance to other stresses and contributes to cell survival under multiple adverse environmental conditions (9, 15, 22). Furthermore, T6SS participates in the recruitment of bacterial outer membrane vesicles (OMVs) in a Pseudomonas quinolone signal (PQS)- or lipopolysaccharide (LPS)-dependent manner, thus facilitating iron acquisition, interbacterial competition, and oxidative stress resistance (14, 24).

Importantly, these functions of T6SS in stress resistance and ion transport were identified to be sensed and responded by certain regulators. In *Burkholderia thailandensis*, OxyR and Zur regulate T6SS4, thus participating in the oxidative stress response (15, 25). In *Yersinia pseudotuberculosis*, RpoS, OmpR, OxyR, ZntR, RelA, and HpaR activate the expression of T6SS4 to resist oxidative and nutrient stress (9, 26–30). In *Pseudomonas aeruginosa*, H3-T6SS is positively regulated by RpoS, thus combating oxidative stress in a DNA-binding proteins from starved cells (DPS)-dependent manner (31). In *Cupriavidus pinatubonensis* (formerly *Alcaligenes eutrophus*, *Ralstonia eutropha*, and *Cupriavidus necator*; 32–34), Fur-regulated T6SS1 is important in iron acquisition with the help of OMVs, facilitating oxidative stress resistance (24); however, the mechanisms by which *C. pinatubonensis* sense and respond to oxidative stress are unknown.

Here, we identified an OxyR ortholog, Reut_A2805, in *C. pinatubonensis*, which positively regulated the expression of T6SS1 by directly binding to its operon promoter region. Further studies revealed that OxyR-regulated T6SS1 promoted iron acquisition in a low-iron environment, thus eliminating the hydroxyl radicals induced by oxidative stress, a process for which the T6SS1-mediated OMV-dependent iron acquisition pathway was essential. Moreover, the T6SS1-mediated OMV-dependent and cupriabactin-mediated siderophore iron acquisition systems are differently regulated by oxidative stress and iron concentration. Our study revealed that the T6SS1 and cupriabactin are coordinated to combat oxidative stress under low-iron conditions, providing new insights into the roles of these two iron-acquisition systems in *C. pinatubonensis*.

## RESULTS

### Reut_A2805 is an OxyR ortholog in *C. pinatubonensis*

**Fig 1:**
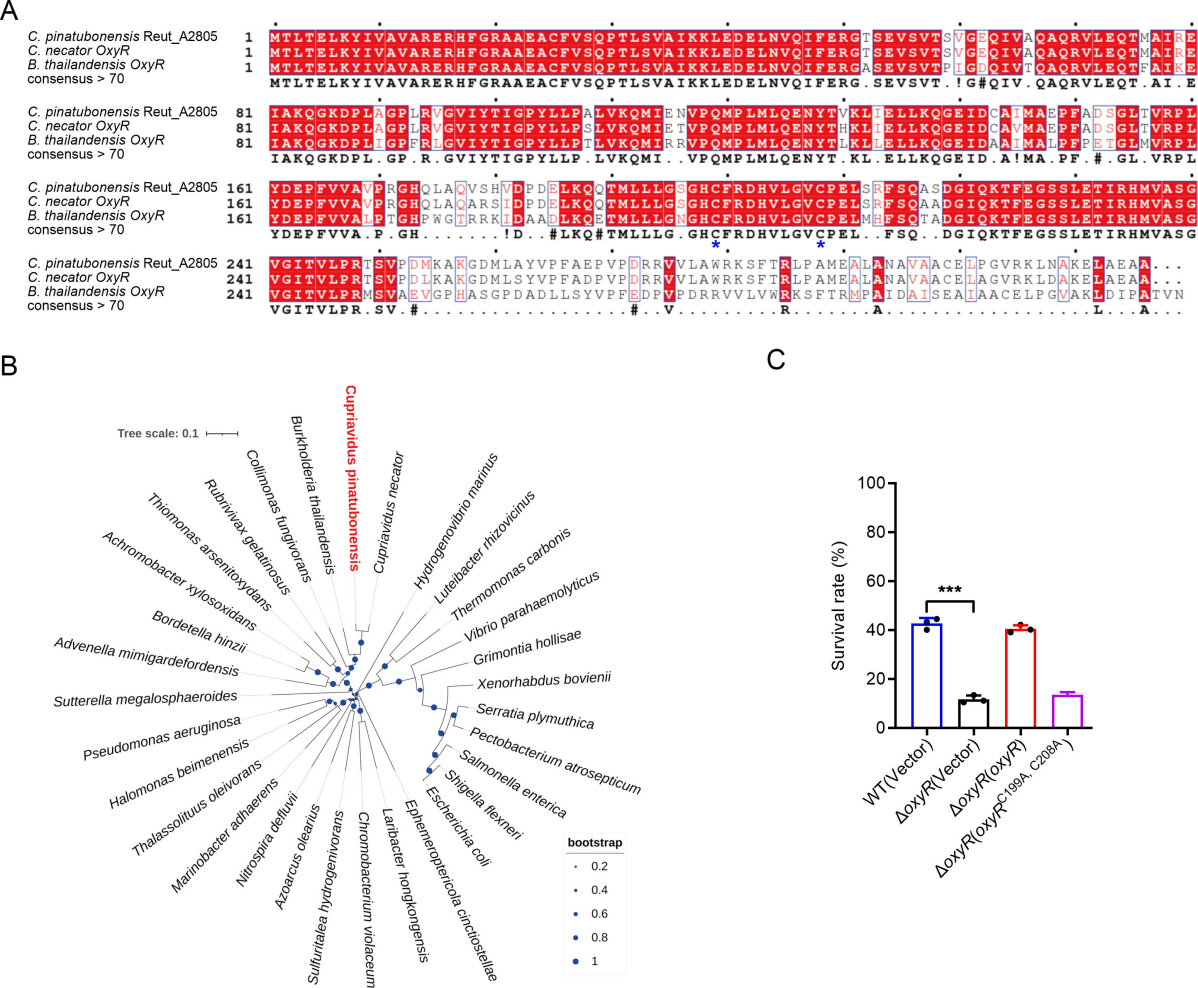
Sequence analysis of OxyR from *C. pinatubonensis*. (**A**) Alignment of Reut_A2805 with OxyR from *C. necator* and *B. thailandensis*. The multiple sequence alignment was performed using ClustalW, and the figure was produced by ESPript. The conserved sequences were colored in red. (**B**) A maximum-likelihood phylogenetic tree generated by the ClustalW alignment of the amino acid sequences of Reut_A2805 and OxyR from different species using MEGA11. The names of microorganisms were listed in the label, and the Protein ID was listed in the Supporting Table S3. The numbers at each node indicate the percentage of 1,000 bootstrap sample whose tree contained the indicated node. The scale bar at the lower left represents a genetic distance of 0.1. (**C**) OxyR is essential for *C. pinatubonensis* to resist oxidative stress. The viability of mid-exponential phase *C. pinatubonensis* strains was determined after exposure to 0.1 mM H_2_O_2_ for 25 min in M9 medium. Data represent the mean ± SD of three biological replicates, each of which was performed with three technical replicates. ***P < 0.001.

OxyR is an oxidative stress-related regulator, but its role in *C. pinatubonensis* has not been characterized. Genomic analysis revealed an OxyR ortholog (Reut_A2805) in *C. pinatubonensis*, the amino acid sequence of which shares 95% and 75% similarity (Fig. 1A) with OxyR in *C. necator* and *B. thailandensis* (22, 35), respectively. The amino acid sequence of Reut_A2805 was aligned with OxyR from other bacterial species using ClustalW, and a phylogenetic tree was generated by MEGA11. Reut_A2805 clustered with other bacterial OxyR proteins (Fig. 1B). Based on a bioinformatics analysis, we annotated the *reut_A2805* gene as the *C. pinatubonensis oxyR* gene and the corresponding protein as OxyR. As a conserved oxidative stress regulator that controls gene expression, OxyR reportedly protects against oxidative stress in bacteria (5, 9, 22). To assess the role of OxyR in resistance to oxidative stress in *C. pinatubonensis*, we constructed an *oxyR* gene deletion mutant and compared its viability with that of the wild type following exposure to H_2_O_2_ for 25 min. The Δ*oxyR* mutant was more susceptible than the WT and the complemented strain upon H_2_O_2_ challenge (Fig. 1C). The C-terminal domain of OxyR in *C. pinatubonensis* contains two redox-active cysteine residues (C199 and C208) that are known to mediate redox-dependent conformational switching in the identified OxyR (36). We simultaneously mutated two cysteine residues and found that the OxyR lacking two redox-active cysteine residues failed to help Δ*oxyR* mutant to resist oxidative stress (Fig. 1C). Therefore, the results all collectively indicated that *reut_A2805* encodes OxyR, which is important in oxidative stress resistance in *C. pinatubonensis*.

### OxyR positively regulates the expression of T6SS1 in *C. pinatubonensis*

**Fig 2:**
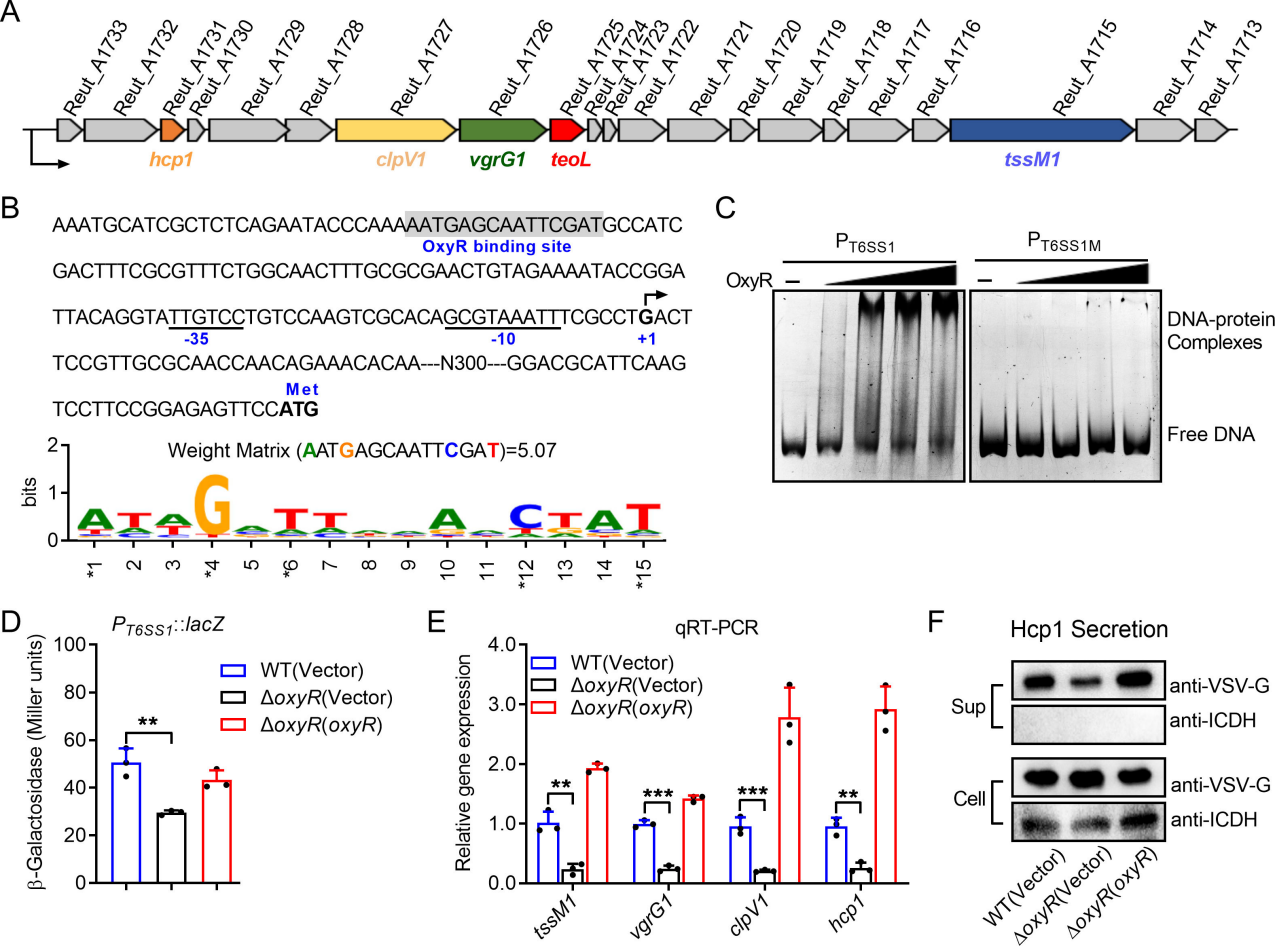
OxyR directly and positively regulates T6SS1 expression in *C. pinatubonensis*. (**A**) Gene organization of the T6SS1 gene cluster in *C. pinatubonensis*. (**B**) Identification of the OxyR binding site in the promoter region of T6SS1. The putative OxyR binding site was identified by the online software Virtual Footprint and indicated by shading, and its sequence has a probability score of 5.07 (max score = 7.01). The Y-axis represents relative nucleotide probability, and the X-axis represents nucleotide position. The ATG start codon of the first ORF of the T6SS1 operon was marked in bold, and the –35 and –10 elements of the T6SS1 promoter are underlined. +1 denotes the transcription start point. (**C**) OxyR binds the T6SS1 promoter. EMSA was performed to analyze the interaction between OxyR with the T6SS1 promoter (P_T6SS1_) and OxyR box mutant DNA (P_T6SS1M_). (**D–F**) OxyR activates the expression of T6SS1. β-galactosidase activity of T6SS1 promoter from chromosomal *lacZ* fusions in relevant *C. pinatubonensis* strains was measured under H_2_O_2_ condition (**D**). Cells of relevant *C. pinatubonensis* strains were grown to stationary phase in M9 medium with 0.02 mM H_2_O_2_, and the expression of *tssM1*, vgrG1, *clpV1*, and *hcp1* (the main components of T6SS1) was measured by quantitative real-time PCR (qRT-PCR) (**E**). The relevant *C. pinatubonensis* strains expressing C-terminal VSV-G-tagged Hcp1 were grown in M9 medium with 0.02 mM H_2_O_2_ to the late logarithmic phase at 30°C. Production (Total) and secretion (Secreted) of Hcp1-VSV-G were detected by immunoblotting using anti-VSV-G antibodies. Isocitrate dehydrogenase (ICDH) was used as a loading control and lysis control for the total and secreted fractions (**F**). Data represent the mean ± SD of three biological replicates, each of which was performed with three technical replicates. **P < 0.01; ***P < 0.001.

As an important transcription factor, OxyR has been reported to regulate T6SS expression, thus helping *Y. pseudotuberculosis* and *B. thailandensis* to resist oxidative stress in a manner associated with metal ion transport (9, 22). We reported that Fur-regulated T6SS1 is important in the acquisition of iron from OMVs for *C. pinatubonensis*, which contributes to bacterial exploitative competition, horizontal gene transfer, and oxidative stress resistance (24). To gain more insight into the functions and regulatory mechanisms of T6SS1 in *C. pinatubonensis*, we analyzed its promoter region using the online software Virtual Footprint. The putative OxyR binding site (AATGAGCAATTCGAT) in *C. pinatubonensis* was highly conserved with OxyR box identified in *Escherichia coli* (37, 38), and its sequence has a probability score of 5.07 (max score = 7.01), which is calculated by applying the position weight matrix to a sequence (Fig. 2A and B). Incubation of the T6SS1 promoter probe [P_T6SS1_, −326 to −502 bp, relative to the ATG start codon of the first open reading frame [ORF] of the T6SS1 operon] with His_6_-OxyR led to the formation of DNA-protein complexes, whereas the unrelated protein bovine serum albumin (BSA) had no effect (Fig. 2C; Fig. S1A in Supporting Information). Furthermore, replacing this 15 bp binding site in the T6SS1 promoter probe with a 15 bp DNA fragment from the *reut_A1727* encoding region abolished the formation of DNA–protein complexes in the electrophoretic mobility shift assay (EMSA) (Fig. 2C), suggesting that OxyR specifically recognizes the promoter region of the T6SS1 operon. The OxyR protein was purified in the absence of high Dithiothreitol (DTT) concentration, which was predominantly regarded as the oxidized form (39). Therefore, we speculated that the oxidized form of OxyR binds to the T6SS1 promoter. As a verification, the effect of OxyR oxidation on the binding was evaluated by EMSA using His_6_-OxyR treated with various concentrations of DTT. As shown in Fig. S1B in Supporting Information, the binding of OxyR to T6SS1 promoter DNA decreased as DTT concentration increased.

To investigate the role of OxyR in the regulation of the T6SS1 operon, a single-copy *P_T6SS1_::lacZ* fusion reporter was introduced into the chromosomes of the WT, Δ*oxyR* mutant, and complemented Δ*oxyR*(*oxyR*) strains, and the LacZ activity of each strain was quantified in M9 medium with H_2_O_2_. Deletion of *oxyR* significantly decreased the activity of the T6SS1 promoter, and this defect was fully restored to the WT level by *oxyR* complementation (Fig. 2D). Next, we verified the positive regulation of T6SS1 by OxyR by quantitative real-time PCR (qRT-PCR) analysis. Deletion of *oxyR* significantly reduced the expression levels of *tssM1*, *vgrG1*, *clpV1*, and *hcp1*; this decrease was completely reversed by the complementation plasmid expressing the regulatory protein OxyR (Fig. 2E). In addition, the positive regulation of OxyR in T6SS1 was confirmed at the protein level. The shuttle plasmid pME6032-*hcp1-vsvg* was constructed and transferred in WT, Δ*oxyR* mutant and Δ*oxyR*(*oxyR*) strains, and the expression of Hcp1-VSV-G was induced by Isopropyl-beta-D-thiogalactopyranoside (IPTG). The secretion of Hcp1 was decreased in the Δ*oxyR* mutant compared to the WT and complemented strains (Fig. 2F). Therefore, OxyR specifically activates T6SS1 expression by binding its promoter in *C. pinatubonensis*.

### OxyR-regulated T6SS1 combats oxidative stress by importing iron under low-iron conditions

**Fig 3:**
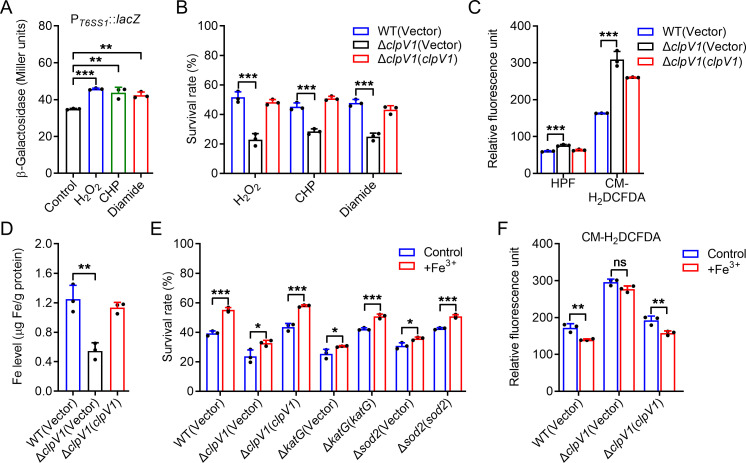
OxyR-regulated T6SS1 combats oxidative stress by importing iron under low-iron conditions. (**A**) T6SS1 expression is induced by oxidative stress under low-iron conditions. Cells of relevant *C. pinatubonensis* strains harboring *P_T6SS1_::lacZ* were grown in M9 medium containing 0.02 mM H_2_O_2_, 0.03 mM CHP, or 0.25 mM diamide, and the LacZ activity was measured. (**B**) T6SS1 is involved in oxidative stress resistance. The viability of *C. pinatubonensis* strains in the mid-exponential phase was determined after exposure to 0.1 mM H_2_O_2_, 0.2 mM CHP, or 1 mM diamide for 25 min in M9 medium. (**C**) Deletion of T6SS1 led to the accumulation of the intracellular ROS under oxidative stress conditions. The intracellular ROS in mid-exponential phase bacterial strains exposed to H_2_O_2_ was determined with the HPF or H_2_DCFDA probe, and fluorescence signals were measured using a microplate reader with excitation/emission wavelengths of 490/515 nm (HPF) and 495/520 nm (CM-H_2_DCFDA). (**D**) Iron uptake requires T6SS1 under oxidative stress conditions. Stationary phase of *C. pinatubonensis* strains was exposed to M9 medium with or without 0.5 mM H_2_O_2_ for 20 min, and the iron associated with bacterial cells was measured by inductively coupled plasma mass spectrometry (ICP-MS). (**E and F**) Alleviation of sensitivity and reduction of intracellular ROS in H_2_O_2_-treated *C. pinatubonensis* strains by exogenous Fe^3+^ required T6SS1. Relevant mid-exponential phase *C. pinatubonensis* strains were exposed to 0.1 mM H_2_O_2_ in M9 medium with or without exogenously provided Fe^3+^ (1 μM) for 25 min. The viability of the cells (**E**) and the intracellular ROS (**F**) was determined, respectively. Data represent the mean ± SD of three biological replicates, each of which was performed with three technical replicates. *P < 0.05; **P < 0.01; ***P < 0.001; ns, not significant.

To investigate whether the expression of OxyR-regulated T6SS1 responds to oxidative stress, we determined the promoter activity of T6SS1. The expression level of T6SS1 was enhanced by the addition of 0.02 mM H_2_O_2_, 0.03 mM CHP, or 0.25 mM diamide under low-iron conditions (Fig. 3A), directly implicating T6SS1 in oxidative stress-resistance. Furthermore, we determined the effect of T6SS1 on bacterial resistance to oxidative stress by measuring viability after exposure to oxidative stress under low-iron conditions. The Δ*clpV1* mutant (lacking conserved T6SS1 structural gene) was significantly more sensitive to oxidative stress (H_2_O_2_, CHP, and diamide) than the WT (Fig. 3B). Meanwhile, the survival rate of the complemented strain was almost completely restored to the WT level (Fig. 3B), supporting a role for T6SS1 in combating oxidative stress under low-iron conditions. Oxidative stress always results in the formation of ROS, which is harmful to living organisms (40). To investigate the effect of T6SS1 on ROS during oxidative stress, we assessed the intracellular ROS levels in *C. pinatubonensis* strains after exposure to H_2_O_2_ using the fluorescent dyes 3′-(*p*-hydroxyphenyl) fluorescein (HPF) and 5-(and-6)-chloromethyl-2′,7′-dichlorodihydrofluorescein diacetate, acetylester (CM-H_2_DCFDA). The Δ*clpV1* mutant had significantly higher ROS levels than the WT after exposure to H_2_O_2_ (Fig. 3C), indicating that T6SS1 is critical for reducing ROS accumulation in *C. pinatubonensis* under oxidative stress conditions. Altogether, these data demonstrated that T6SS1 is induced and involved in oxidative stress resistance under low-iron conditions.

*C. pinatubonensis* T6SS1 is involved in iron acquisition, as indicated by the missing major iron transport systems FeoABC and cupriabactin siderophore under low-iron conditions (24). To test whether the increased T6SS1-dependent survival under oxidative stress conditions is due to iron acquisition, we measured the total metal content in bacterial cells treated with H_2_O_2_ under low-iron conditions using inductively coupled plasma mass spectrometry (ICP-MS). Deletion of *clpV1* significantly reduced the intracellular iron level and complementation of *clpV1* reversed the defect (Fig. 3D). By contrast, the accumulation of other metal ions, such as sodium and magnesium, was not affected in the Δ*clpV1* mutant strain (Fig. S2 in Supporting Information), suggesting that the T6SS1 of *C. pinatubonensis* is associated with iron acquisition under low-iron and oxidative stress conditions.

To verify that the oxidative stress resistance of T6SS1 is related to intracellular iron, a low concentration of Fe^3+^ was supplied during H_2_O_2_ treatment. Whereas exogenous Fe^3+^ (1 µM) markedly increased the survival rate of the WT and complemented strains Δ*clpV1*(*clpV1*) under H_2_O_2_ challenge, the protective effect was largely abolished in the Δ*clpV1* mutant (Fig. 3E). Fe-associated proteins including catalase and superoxide dismutase were used as controls because they are crucial for mitigatingROS-related stress (41, 42). As expected, exogenous Fe^3+^ markedly restored the growth of *C. pinatubonensis* in H_2_O_2_ in the presence of the antioxidant Sod2 or KatG (Fig. 3E). Additionally, exogenous Fe^3+^ (1 µM) significantly decreased the intracellular ROS level in the WT and complemented Δ*clpV1*(*clpV1*) strains (Fig. 3F). These results suggested a role for T6SS1 in importing iron from the environment under low-iron conditions in the presence of oxidative stress.

### T6SS1-mediated OMV-dependent iron acquisition is essential for *C. pinatubonensis* survival under low-iron and oxidative conditions

**Fig 4:**
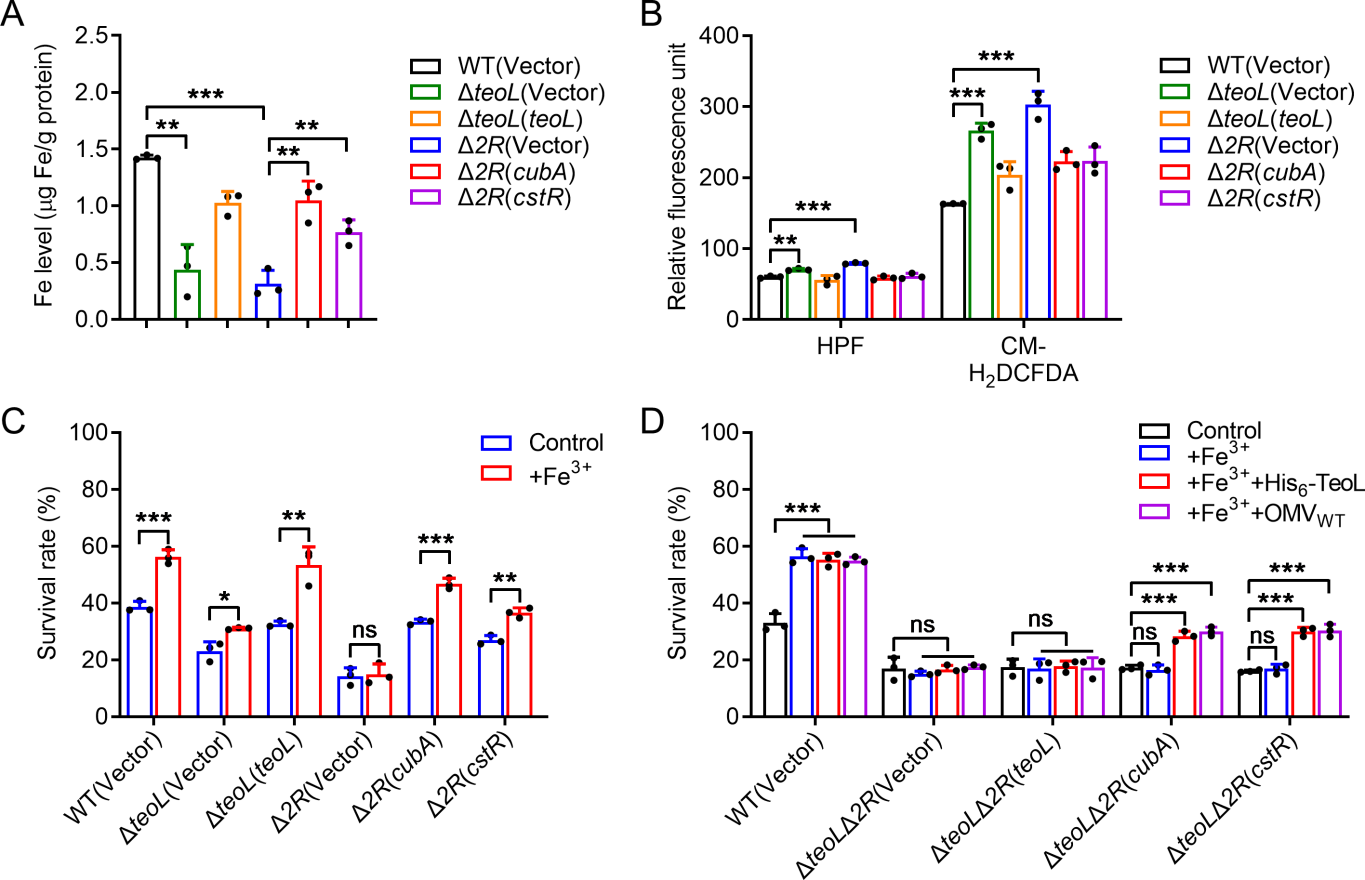
T6SS1-mediated OMV-dependent iron acquisition is essential for *C. pinatubonensis* survival under oxidative stress. (**A**) The T6SS1-mediated OMV-dependent iron acquisition is important for the accumulation of intracellular iron under oxidative stress conditions. Stationary phase of *C. pinatubonensis* strains was exposed to 0.5 mM H_2_O_2_ in M9 medium for 20 min, and the iron associated with bacterial cells was measured by ICP-MS. (**B**) Reduction of intracellular ROS in H_2_O_2_-treated *C. pinatubonensis* strains requires the T6SS1-mediated OMV-dependent iron acquisition. The intracellular ROS in mid-exponential phase bacterial strains exposed to H_2_O_2_ was determined with the HPF or CM-H_2_DCFDA probe, and fluorescence signals were measured using a microplate reader. (**C**) Alleviation of the sensitivity of *C. pinatubonensis* strains to H_2_O_2_ by exogenous Fe^3+^ requires T6SS1-mediated OMV-dependent iron acquisition. Relevant mid-exponential phase *C. pinatubonensis* strains were exposed to 0.1 mM H_2_O_2_ in M9 medium with or without exogenously provided Fe^3+^ (1 μM) for 25 min, and the viability of the cells was determined. (**D**) The T6SS1-mediated OMVs recruitment system contributes to oxidative stress resistance. Relevant mid-exponential phase *C. pinatubonensis* strains were exposed to 0.1 mM H_2_O_2_ in M9 medium containing Fe^3+^ (1 μM), Fe^3+^ (1 μM) + apo-TeoL protein (1 μM), or Fe^3+^ (1 μM) + OMV_WT_ (20 µg mL^−1^ of phospholipids) for 25 min, and the viability of the cells was determined. Data represent the mean ± SD of three biological replicates, each of which was performed with three technical replicates. *P < 0.05; **P < 0.01; ***P < 0.001; ns, not significant.

*C. pinatubonensis* has an iron acquisition pathway consisting of T6SS1-secreted LPS-binding effector TeoL, and TonB-dependent outer membrane receptors CubA and CstR, which contributes to the acquisition of iron in Δ*cubE*Δ*feoB* (Δ*2Fe*) mutant background, which is defective in both cupriabactin and FeoABC iron transport systems (24). To explore the role of T6SS1-mediated OMV recruitment in bacterial iron acquisition and oxidative stress resistance, we determined the total metal contents in bacterial cells treated with H_2_O_2_ using ICP-MS under low-iron conditions. The Δ*teoL* and Δ*cubA*Δ*cstR* (Δ*2R*) mutants exhibited significantly reduced intracellular iron levels compared to the WT and Δ*teoL*(*teoL*), Δ*2R*(*cubA*), and Δ*2R*(*cstR*) complemented strains (Fig. 4A). The accumulation levels of other metal ions, such as sodium and magnesium, were unaffected (Fig. S3 in Supporting Information), implicating T6SS1-mediated OMV recruitment in intracellular iron accumulation under oxidative stress conditions. Furthermore, we assessed the intracellular ROS levels in *C. pinatubonensis* WT, Δ*teoL*, and Δ*2R* mutant strains challenged with H_2_O_2_. Like Δ*clpV1*, the Δ*teoL* and Δ*2R* mutants had significantly higher ROS levels than the WT after exposure to H_2_O_2_, and their respective complemented strains recovered the reduced ROS levels (Fig. 4B), indicating that reduction of intracellular ROS in H_2_O_2_-treated strains requires the T6SS1-mediated OMV-dependent iron acquisition pathway.

The above results prompted us to examine whether this pathway is involved in iron acquisition. Indeed, the Δ*teoL* and Δ*2R* mutant strains were more sensitive to H_2_O_2_ than the WT and complemented strains (Fig. 4C). Importantly, exogenous Fe^3+^ (1 µM) enhanced the survival rate of the WT and complemented strains exposed to H_2_O_2_, whereas the effect of exogenous Fe^3+^ was largely abolished in the Δ*teoL* or Δ*2R* mutant strain (Fig. 4C). We next constructed a Δ*teoL*Δ*2R* mutant, which has deficits in TeoL production and OMV recruitment. The survival rates of Δ*teoL*Δ*2R* and the corresponding single-gene complemented strains were determined following exposure to H_2_O_2_ for 25 min, in the presence of Fe^3+^, Fe^3+^+His_6_-TeoL, or Fe^3+^+OMV_WT_. Although His_6_-TeoL and OMVs purified from the WT significantly increased the survival rate of the WT strain, they had no effect on the Δ*teoL*Δ*2R* mutant, indicating that the ability to obtain OMVs is crucial for resisting oxidative stress (Fig. 4D). Moreover, adding OMVs purified from the WT strain substantially improved the survival rates of Δ*teoL*Δ*2R* complemented with the OMV receptor gene *cubA* or *cstR* but not *teoL* (Fig. 4D). The results suggested that the T6SS1-mediated OMV-dependent iron acquisition pathway is essential for *C. pinatubonensis* survival under oxidative stress.

### Two iron acquisition systems are coupled to combat oxidative stress

**Fig 5:**
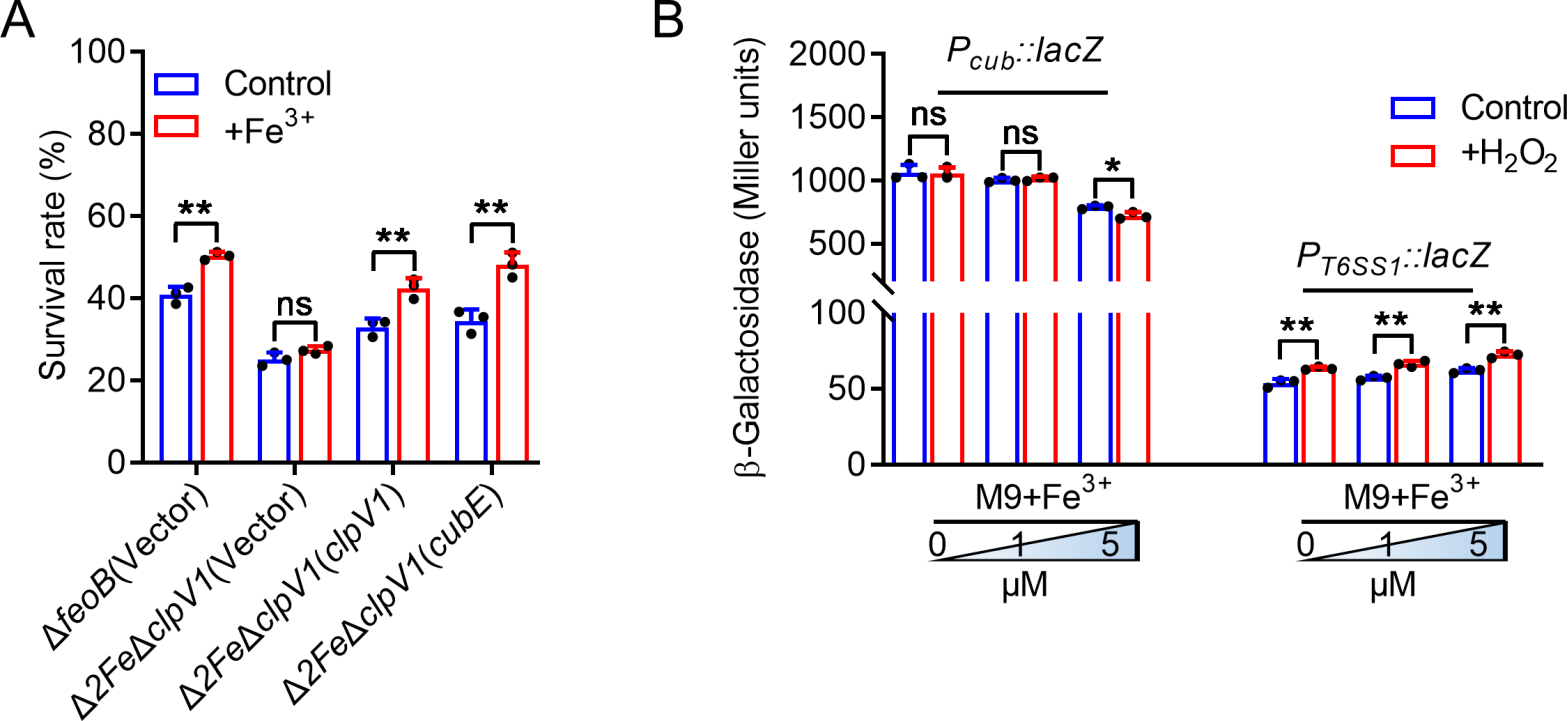
Two iron acquisition systems are differently induced and coupled to combat oxidative stress. (**A**) Alleviation of the sensitivity of *C. pinatubonensis* strains to H_2_O_2_ by exogenous Fe^3+^ requires two iron acquisition systems mediated by T6SS1 and cupriabactin. Relevant mid-exponential phase *C. pinatubonensis* strains were exposed to 0.1 mM H_2_O_2_ in M9 medium with or without exogenously provided Fe^3+^ (1 μM) for 25 min, and the viability of the cells was determined. (**B**) Expression of *cub* and *T6SS1* is affected by iron concentrations and oxidative stress, respectively. β-galactosidase analysis of *cub* and *T6SS1* promoter activities was performed in *C. pinatubonensis* strains grown to stationary phase in M9 medium with or without 0.02 mM H_2_O_2_ containing different concentrations of Fe^3+^ (0, 1, and 5 μM). Data represent the mean ± SD of three biological replicates, each of which was performed with three technical replicates. *P < 0.05; **P < 0.01; ns, not significant.

In *C. pinatubonensis*, we identified two types of iron acquisition systems mediated by T6SS1 and the siderophore cupriabactin (24, 43). To determine the antioxidant mechanism of these two iron acquisition systems, we generated a Δ*2Fe*Δ*clpV1* mutant in the background of the Δ*feoB* strain defective in FeoABC iron transport systems. Although the sensitivity to H_2_O_2_ of the Δ*2Fe*Δ*clpV1* mutant (lacking T6SS1 and cupriabactin) was not rescued by exogenous Fe^3+^, the sensitivity of strains Δ*2Fe*Δ*clpV1*(*clpV1*) and Δ*2Fe*Δ*clpV1*(*cubE*) was rescued by exogenous Fe^3+^ (Fig. 5A), suggesting that T6SS1- and cupriabactin-mediated importation of Fe^3+^ is crucial in oxidative stress resistance. To explore the regulation of these two iron-acquisition systems, we first determined the effect of OxyR on the expression of the cupriabactin system by measuring the transcription levels of chromosomal *P_cub_::lacZ* fusions. The expression of *cub* was not significantly affected in the *C. pinatubonensis* Δ*oxyR* mutant (Fig. S4 in Supporting Information), indicating that OxyR does not directly regulate the cupriabactin siderophore system. Next, we analyzed the expression of *C. pinatubonensis cub* and *T6SS1* in M9 medium with or without 20 µM H_2_O_2_ containing Fe^3+^ (0, 1, or 5 µM), respectively. *Cub* expression was markedly repressed by 5 µM Fe^3+^, and H_2_O_2_ intensified the inhibitory effect (Fig. 5B), suggesting that the *cub* system functions to combat oxidative stress is iron concentration-dependent. By contrast, the expression of T6SS1 was increased by low iron (1 and 5 µM). Moreover, H_2_O_2_ further activated its expression under low-iron conditions (0, 1, and 5 µM; Fig. 5B), suggesting that the T6SS1 iron acquisition system is actively induced to cope with oxidative stress under low-iron conditions. Altogether, these data indicated that two iron acquisition systems have different mechanisms and are essential for bacterial resistance to oxidative stress under low-iron conditions.

## DISCUSSION

The T6SS1 of *C. pinatubonensis* was reported to be negatively regulated by Fur, an iron transport-related repressor, which participates in the acquisition of iron, enabling bacteria to combat oxidative stress (24). However, the mechanism of oxidative stress resistance was unclear. In this study, we identified Reut_A2805 as an OxyR ortholog in *C. pinatubonensis*, and an Δ*oxyR* mutant showed a significant defect in oxidative stress resistance (Fig. 1). OxyR, as a conserved oxidative stress regulator that controls gene expression, is important in protection against oxidative stress in several bacterial taxa (5, 9, 22). Then, the OxyR positive regulates the expression of T6SS1 (Fig. 2), suggesting that the oxidative resistance of T6SS1 is facilitated by this regulator. The expression of the OxyR-regulated T6SS1 responded to oxidative stress, relieving the intracellular ROS accumulation induced by oxidative stress (Fig. 3). Also, T6SS1-mediated iron acquisition significantly enhanced bacterial resistance to oxidative stress (Fig. 3) in a manner dependent on the OMV-dependent iron acquisition pathway (Fig. 4). Our findings reveal the mechanism of *C. pinatubonensis* T6SS1 in combating oxidative stress by which bacteria sense and respond to oxidative stress via its positive regulator OxyR.

Iron has been implicated in many oxidative stress-related pathways and conditions, for it is a cofactor of many antioxidant enzymes, such as catalase and superoxide dismutase. It is also a primary cause of ROS generation via the Fenton reaction (44). Maintaining intracellular iron homeostasis is particularly important for bacterial survival. Fur, as a well-documented iron-dependent repressor, controls the switch of the iron transport system. The T6SS1 and cub systems are negatively regulated by Fur, facilitating iron acquisition under low-iron conditions, and promoting resistance to oxidative stress (24, 43). However, the mechanisms were unclear. In this study, we determined the mechanism of oxidative stress resistance and evaluated the underlying mechanism under low-iron conditions in the presence of oxidative stress. T6SS1 expression responded to oxidative stress and was positively regulated by OxyR, thus enhancing oxidative stress resistance via iron acquisition, while *cub* expression was strictly iron concentration-dependent and repressed by oxidative stress (Fig. 5). We infer that the T6SS1-mediated iron acquisition system functions in oxidative resistance is driven by oxidative stress under low-iron conditions, while the antioxidative capacity of *cub*-mediated iron acquisition system functions only because of the low iron concentration.

OMV, as a unique and versatile secretion system, participates in multiple biological processes by delivering varied biologically active molecules at high concentrations to distant bacterial or mammalian cells (45). The ability of OMV to resist oxidative stress is nascently demonstrated, and the mechanism is unclear. The identification of iron in *P. aeruginosa* and *C. pinatubonensis* OMVs (14, 24) and catalase in *Helicobacter pylori* OMVs (46) supports their role in oxidative stress resistance, which is consistent with the role of vesiculation in the oxidative stress response (47). In this study, we evaluated the relationships among iron, OMV, T6SS1, and siderophore under low-iron conditions in the presence of oxidative stress. As a dominant siderophore that mainly responds to the iron uptake in *C. pinatubonensis*, cupriabactin still proceeded to acquire iron in response to iron deficiency under oxidative stress conditions. Thus, its strategy is to slow down the rate of iron acquisition by decreasing the activity of the *cub* promoter. As a nonspecific iron acquisition system, the oxidative stress-induced T6SS1 and OMV were coupled to acquire iron, as well as other cargoes, and the bacterial iron uptake rate is relatively decreased. Hence, the strategy of T6SS1 and OMVs is further to decelerate the iron acquisition, and antioxidase may function in this process.

**Fig 6:**
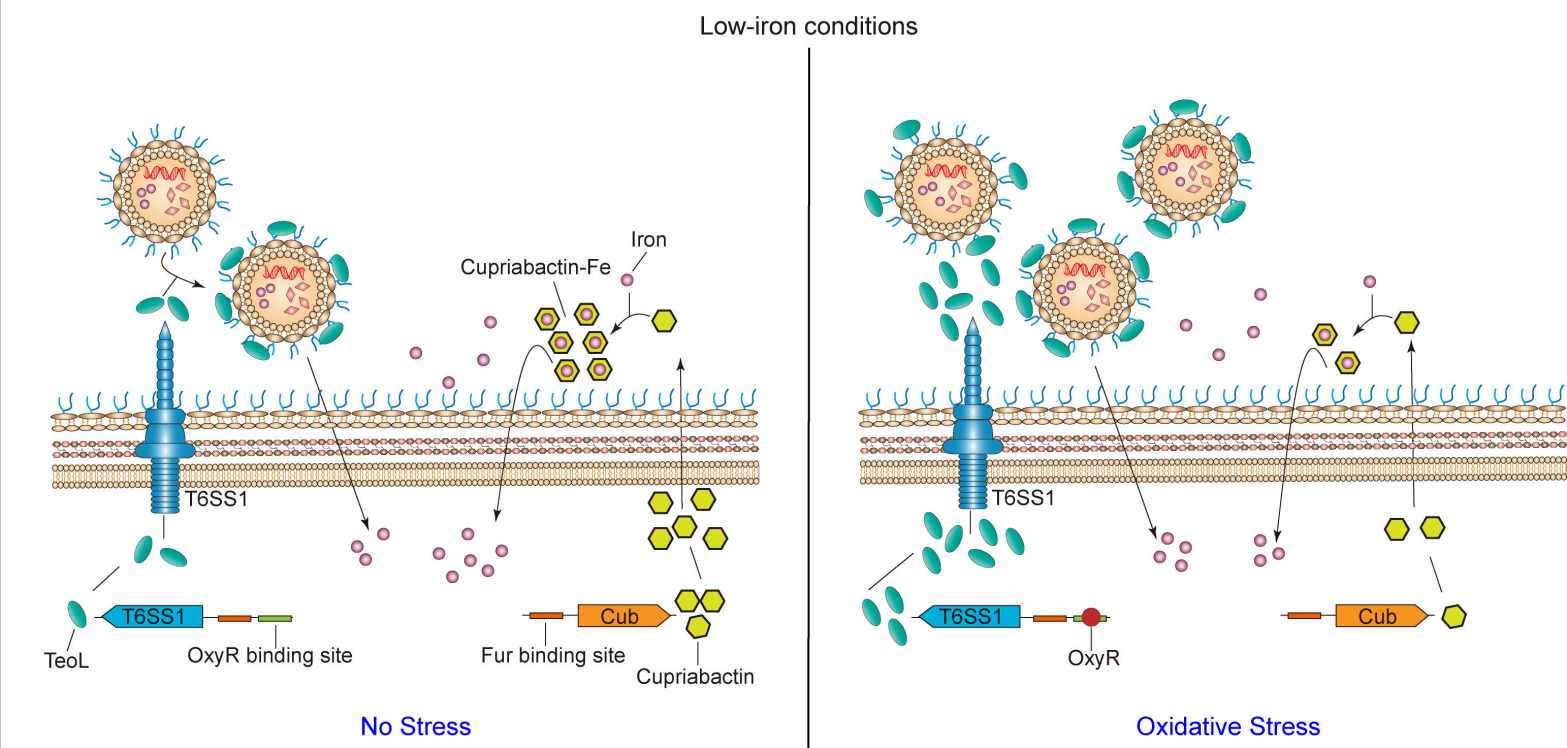
Model of T6SS1- and siderophore-mediated iron acquisition under low-iron and oxidative stress conditions in *C. pinatubonensis*. Under low-iron conditions, the Fur-repressed *T6SS1* and *cub* gene clusters are de-repressed, and both facilitate the iron acquisition for *C. pinatubonensis* by recruiting OMV and secreting siderophore cupriabactin, respectively. Under this condition, *cub* operon is highly expressed and mainly responsible for the iron uptake, while T6SS1 is lowly expressed and functions weakly. Upon encountering oxidative stress, bacteria suppress iron acquisition by decreasing *cub* promoter activity and inducing T6SS1 expression by OxyR, in which iron is acquired in a nonspecific manner, thereby promoting resistance to oxidative stress.

Based on our results, we unravel a complex iron acquisition pathway involving T6SS and siderophore iron transport systems that enhances bacterial survival under low-iron and oxidative stress conditions (Fig. 6). Under low-iron conditions, the Fur-repressed *T6SS1* and *cub* gene clusters are de-repressed, thereby promoting iron acquisition in *C. pinatubonensis* by recruiting OMVs and secreting cupriabactin, respectively. Under this condition, the *cub* operon is highly expressed and mainly responsible for iron uptake, whereas T6SS1 is expressed at a low level. Upon encountering oxidative stress, bacteria suppress iron acquisition by decreasing *cub* promoter activity and inducing T6SS1 expression by OxyR, in which iron is acquired in a nonspecific manner, thereby promoting resistance to oxidative stress. Overall, our findings not only reveal the mechanism by which T6SS1 enhances resistance to oxidative stress but also demonstrate how the T6SS1 and cub iron acquisition systems sense and respond to oxidative stress under low-iron conditions in *C. pinatubonensis*, providing a clear perspective for understanding the bacterial iron acquisition in multiple complicated environments.

## MATERIALS AND METHODS

### Bacterial strains and growth conditions

The bacterial strains and plasmids used in this study are listed in Table S1 in Supporting Information. *E. coli* strains were grown in Luria Bertani (LB) broth with appropriate antibiotics at 37°C. The *C. pinatubonensis* strains were cultured in Nutrient broth (NB) or M9 minimal medium (48) (Na_2_HPO_4_, 6 g L^−1^; KH_2_PO_4_, 3 g L^−1^; NaCl, 0.5 g L^−1^; NH_4_Cl, 1 g L^−1^; MgSO_4_, 2 mM; CaCl_2_, 0.1 mM; glucose 0.2%, pH 7.0) at 30°C with appropriate antibiotics when necessary. All chemicals were of analytical reagent grade purity or higher. Antibiotics were added at the following concentrations: kanamycin, 50 µg mL^−1^; gentamycin, 10 µg mL^−1^; tetracycline, 20 µg mL^−1^.

### Plasmid construction

The primers used in this study are listed in Table S2 in Supporting Information. The plasmid pK18-Δ*oxyR* (*reut_A2805*) was used to construct the Δ*oxyR* in-frame deletion mutant of *C. pinatubonensis*. The primer pairs *oxyR*-1F-EcoRI/*oxyR*-1R and *oxyR*-2F/*oxyR*-2R-SalI were used to amplify a 756 bp upstream segment and a 910 bp downstream segment of the *oxyR*, respectively. The upstream and downstream PCR fragments were ligated by overlapping PCR. The resulting PCR products were digested with EcoRI/SalI and inserted into the EcoRI/SalI sites of pK18 to produce pK18-Δ*oxyR*. The knock-out plasmids pK18-Δ*katG* (*reut_A1741*) and pK18-Δs*od2* (*reut_A0597*) were constructed in a similar method by using primers list in Table S2 in Supporting Information.

To complement the Δ*oxyR* mutant, primers *oxyR-*F-KpnI/*oxyR-*R-SacI were used to amplify the *oxyR* gene from the *C. pinatubonensis* genome DNA. The PCR product of *oxyR* was digested with KpnI/SacI and inserted into the KpnI/SacI sites of pBBR1MCS-5 to produce pBBR1MCS-5-*oxyR*. The complementation plasmids pBBR1MCS-2-*katG* and pBBR1MCS-2-*sod2* were constructed in a similar method by using primers list in Table S2 in Supporting Information. To express His_6_-tagged OxyR, the plasmid pET28a-*oxyR* was constructed. Briefly, the primers *oxyR-*F-EcoRI and *oxyR-*R-SalI were used to amplify the *oxyR* gene fragment from the *C. pinatubonensis* genome. The PCR product of *oxyR* was digested with EcoRI/SalI and inserted into the BamHI/SalI sites of pET28a to generate pET28a-*oxyR*. The shuttle plasmid pME6032-*hcp1-vsvg* was constructed in a similar method.

For complementation, the complementary plasmids pBBR1MCS-5-*oxyR*, pBBR1MCS-2-*katG*, and pBBR1MCS-2-*sod2* were introduced into the respective mutants by electroporation. The integrity of the insert in all constructions was confirmed by DNA sequencing.

### Overexpression and purification of recombinant protein

To express and purify soluble His_6_-tagged recombinant proteins, the plasmid pET28a- *oxyR* was transformed into BL21(DE3). Bacteria were cultured at 37°C in LB medium to an OD_600_ of 0.5, shifted to 24°C and induced with 0.2 mM IPTG, and then cultivated for an additional 12 h at 24°C. Harvested cells were disrupted by sonication, and proteins were purified with the His•Bind Ni-NTA resin (Novagen, Madison, WI) according to the manufacturer’s instructions. Eluted recombinant proteins were dialyzed against buffer (50 mM Tris, 150 mM NaCl, 10% glycerol, and pH 7.5) at 4°C. The resulting proteins were stored at −80°C until use. Protein concentrations were determined using the Bradford assay according to the manufacturer’s instructions (Bio-Rad, Hercules, CA) with bovine serum albumin as standard.

### Electrophoretic mobility shift assay

EMSA was performed by Zhang and colleagues (49). The *P_T6SS1_* fragment was amplified from the *C. pinatubonensis* genome using the primers *T6SS1*-EMSA-F and *T6SS1*-EMSA-R. Increasing concentrations of purified His_6_-OxyR (0.01, 0.05, 0.10, and 0.15 µM) were incubated with 5 ng DNA probes in EMSA buffer (20 mM Tris, pH 7.4, 4 mM MgCl_2_, 100 mM NaCl, and 10% glycerol). After incubation for 20 min at room temperature, the binding reaction mixture was subjected to electrophoresis on a 6% native polyacrylamide gel containing 5% glycerol in 0.5 × TBE (Tris-borate-EDTA) electrophoresis buffer, and the DNA probe was detected using SYBR Green. As negative controls, irrelevant protein bovine serum albumin was included in the binding assay.

### Construction of chromosomal fusion reporter strain and β-galactosidase assay

The *lacZ* fusion reporter vector pK18-P*_T6SS1p_::lacZ* was transformed into *E. coli* S17-1 *λ pir* and mated with *C. pinatubonensis* strains as described previously (43). The *lacZ* fusion reporter strains were grown to stationary phase in NB or M9 medium at pH 7.0 under 30°C unless otherwise specified, and β-galactosidase activity was assayed using ONPG (o-Nitrophenyl β-D-galactopyranoside) as the substrate. These assays were performed in triplicate at least three times, and the error bars represent SDs.

### Quantitative real-time PCR

Bacteria cells were harvested during the mid-exponential phase, and RNA was extracted using the RNAprep Pure Cell/Bacteria Kit and treated with RNase-free DNase (TIANGEN, Beijing, China). The purity and concentration of the RNA were determined by gel electrophoresis and spectrophotometer (NanoDrop, Thermo Scientific). First-strand cDNA was reverse transcribed from 1 µg of total RNA with the TransScript First-Strand cDNA Synthesis SuperMix (TransGen Biotech, Beijing, China). qRT-PCR was performed in CFX96 Real-Time PCR Detection System (Bio-Rad, USA) with TransStart Green qPCR SuperMix (TransGen Biotech, Beijing, China). For all primer sets (Table S2 in Supporting Information), the following cycling parameters were used: 95°C for 30 s followed by 40 cycles of 94°C for 15 s and 55°C for 30 s. For standardization of the results, the relative abundance of 16S rRNA was used as the internal standard. All samples were analyzed in triplicate, and the expression of target genes was calculated as relative fold values using the 2^-ΔΔCT^ method. These assays were performed in triplicate at least three times, and the error bars represent the SEM.

### Protein secretion assay

The shuttle plasmid pME6032-*hcp1-vsvg* was constructed and transferred in relevant strains, and the expression of Hcp1-VSV-G was induced by IPTG. Secretion assay for Hcp1 was performed according to described methods (50). Briefly, strains were inoculated into 100 mL NB and incubated with continuous shaking until OD_600_ reached 1.5 at 30°C. 1 mL of culture was centrifuged, and the cell pellet was resuspended in 100 µL Sodium dodecyl sulfate (SDS)-loading buffer; the whole-cell lysate sample was defined as Hcp1_cells_. 80 mL of the culture was centrifuged, and the supernatant was filtered through a 0.22 µm filter (Millipore, MA, USA). The secreted proteins in the supernatant were collected by filtration over a nitrocellulose filter (BA85) (Whatman, Germany) for three times. The filter was soaked in 100 µL SDS sample buffer for 15 min at 65°C to recover the proteins present, and the sample was defined as Hcp1_sup_. All samples were normalized to the OD_600_ of the culture and volume used in preparation.

### Western blot analysis

Samples were resolved by SDS-Polyacrylamide gel electrophoresis (PAGE) and transferred onto Polyvinylidene difluoride (PVDF) membranes (Millipore). The membrane was blocked with QuickBlock Blocking Buffer (Beyotime Biotechnology, Haimen, China) for 30 min at room temperature and incubated with primary antibodies at 4°C overnight: anti-VSV-G (Santa Cruz biotechnology, USA), 1:500; anti-ICDH, 1:6000; the membrane was washed three times in Triethanolamine-buffered saline and tween (TBST) buffer (50 mM Tris, 150 mM NaCl, 0.05% Tween 20, and pH 7.4) and incubated with 1:5,000 dilution of horseradish peroxidase-conjugated secondary antibodies (Beyotime Biotechnology, Haimen, China) for 1 h. Signals were detected using the ECL kit (Invitrogen) following the manufacturer’s specified protocol.

### Bacterial survival assay

Mid-exponential phase *C. pinatubonensis* strains grown in NB medium were collected, washed, diluted 50-fold into M9 medium, and treated with H_2_O_2_ (0.1 mM), CHP (0.03 mM), or diamide (0.25 mM), respectively, at 30°C for 25 min. After treatment, the cultures were serially diluted and plated onto NB agar plates, and colonies were counted after 48 h growth at 30°C. Percentage survival was calculated by dividing the number of Colony forming units (CFU) of stressed cells by the number of CFU of cells without stress. All these assays were performed in triplicate at least three times.

### OMV isolation, purification, and quantification

OMVs were isolated, purified, and quantified as described (24). Briefly, to obtain OMVs without bacterial cells, overnight batch culture was centrifuged for 20 min at 6,000 × *g* and 4°C. The supernatant was filtered through 0.45 and 0.22 µm vacuum filter, respectively, to thoroughly remove the remaining bacteria. The resulting filtrate was ultracentrifuged for 1 h at 200,000 × g at 4°C using an angle rotor (70 Ti, Beckman Coulter, USA), and the pellets were washed twice with phosphate-buffered saline (PBS), which were subsequently resuspended in 50 mM HEPES-0.85% NaCl. For purification, crude OMV samples were adjusted to 1 mL of 45% (wt/vol) iodixanol (OptiPrep; Sigma-Aldrich) in HEPES-NaCl, transferred to the bottom of ultracentrifuge tubes, and layered with iodixanol-HEPES-NaCl (2 mL of 40, 35, 30, 25, and 20%). The samples were ultracentrifuged for 4 h at 150,000 × *g* at 4°C using a swing rotor (SW40 Ti, Beckman Coulter, USA). Then, 1 mL fractions were collected from each gradient and detected by SDS-PAGE. The fraction containing OMV was ultracentrifuged for 1 h at 200,000 × *g* at 4°C using an angle rotor and resuspended in HEPES-NaCl. For quantification, the protein concentration and the phospholipid concentration of the OMV were measured, respectively.

### Fluorescence dye-based intracellular ROS detection

To detect intracellular ROS, the fluorescent reporter dyes 3’-(p-hydroxyphenyl) fluorescein (HPF, Invitrogen) and 5-(and-6)-chloromethyl-2’,7’-dichlorodihydrofluorescein diacetate (CM-H_2_DCFDA, Invitrogen) were used as previously described (51). Briefly, 1 mL samples were collected, washed with PBS, and then resuspended in 1 mL of M9 containing 10 µM HPF or CM-H_2_DCFDA, respectively. Samples were incubated in the dark for 20 min at 30°C. The cells were then pelleted, the supernatant removed, and resuspended in 1 mL M9 medium containing 0.4% glucose with or without H_2_O_2_ (0.02 mM). After 25 min of treatment at 30°C, the cells were pelleted, washed with PBS, and resuspended in 1 mL of M9, and then, 200 µL of the resultant cell suspension were transferred to a dark 96-well plate. Fluorescence signals were measured using a SpectraMax M2 Plate Reader (Molecular Devices) with excitation/emission wavelengths of 495/520 nm (HPF) and 495/520 nm (CM-H_2_DCFDA).

### Determination of intracellular ion contents

Intracellular ion content was determined as described previously (50). Briefly, cells were grown in M9 minimal medium until mid-exponential phase. After 20 mL culture solutions were collected and washed with M9 for two times, the pellets were re-dissolved in 20 mL M9 medium containing 0.5 mM H_2_O_2_ and then incubated further for 25 min. The cell pellet weight was measured, and bacteria were chemically lysed using Bugbuster (Novagen, Madison, WI) according to the manufacturer’s instructions. Bacteria were resuspended in Bugbuster solution by pipetting and incubation on a rotating mixer at a slow setting for 12 h. Total protein for each sample was measured by using NanoDrop ND-1000 spectrophotometer (NanoDrop Technologies) according to the manufacturer’s instructions. Each sample was diluted 10-fold in 2% molecular grade nitric acid to a total volume of 5 mL at a slow setting for 12 h. Samples were analyzed by ICP-MS (Varian 802 MS), and the result was corrected using the appropriate buffers for reference and dilution factors. Triplicate cultures of each strain were analyzed during a single experiment, and the experiment was repeated at least three times.

### Bioinformatics analysis

Sequence alignment and database searches were carried out using the BLAST server of the National Center for Biotechnology Information (NCBI) website (https://www.ncbi.nlm.nih.gov/) and visualized by using DNAMAN software. The multiple sequence alignments were performed using ClustalW, and the phylogenetic tree was generated by MEGA11.

### Statistical analysis

Experimental data analyzed for significance were performed by using GraphPad Prism 8 (GraphPad Software, San Diego California USA). Statistical analysis for the rest of the assays was performed using unpaired two-tailed Student’s *t* test. Error bars represent ±SD. *P < 0.05; **P < 0.01; ***P < 0.001.

## Data Availability

All datasets generated for this study are included in the article/Supporting Information.

## SUPPLEMENTAL MATERIAL

Supplemental material spectrum.03231-23-s0001.docx Supplemental tables and figures.

ASM does not own the copyrights to Supplemental Material that may be linked to, or accessed through, an article. The authors have granted ASM a non-exclusive, world-wide license to publish the Supplemental Material files. Please contact the corresponding author directly for reuse.
