# Supplementary material for: OxyR-regulated T6SS functions in coordination with siderophore to resist oxidative stress
Source: Microbiol Spectr. 2024 Jan 8;12(2):e03231-23. doi: 10.1128/spectrum.03231-23 (PMC10846153; doi:10.1128/spectrum.03231-23)
Supplement: Supplemental material — Supplemental tables and figures. [file spectrum.03231-23-s0001.docx]

**Supporting Information**

**OxyR-regulated T6SS functions in coordination with siderophore to resist oxidative stress**

**This file includes:**

**Supporting Figures 1-5**

**Supporting Tables 1-3**

**Supporting References**


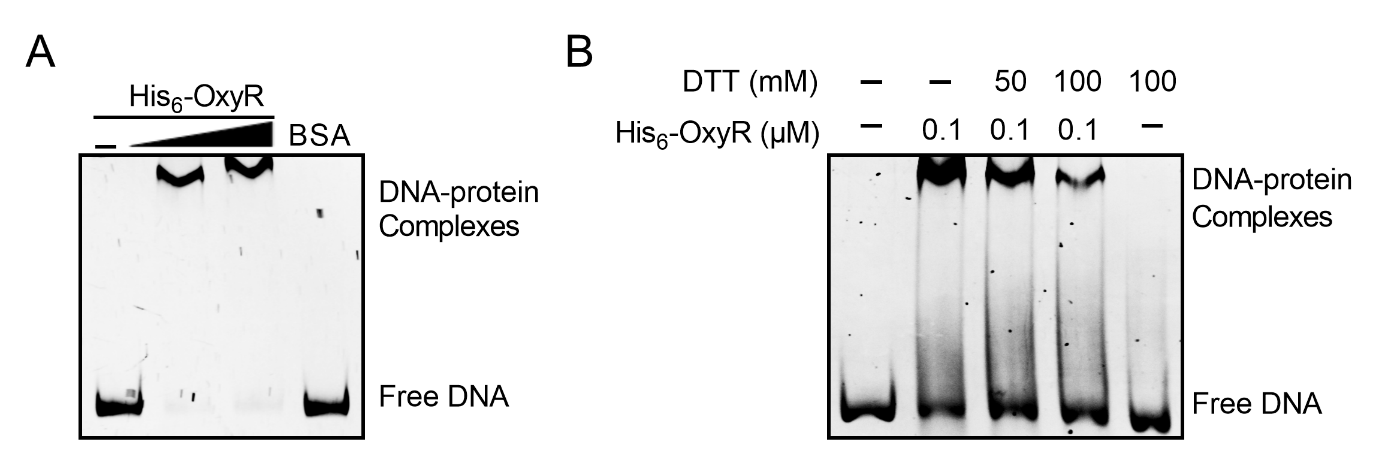


# Figure S1 OxyR binds the T6SS1 promoter. EMSA was performed to analyze the interaction between OxyR and the T6SS1 promoter (P_T6SS1_). BSA was a negative control.


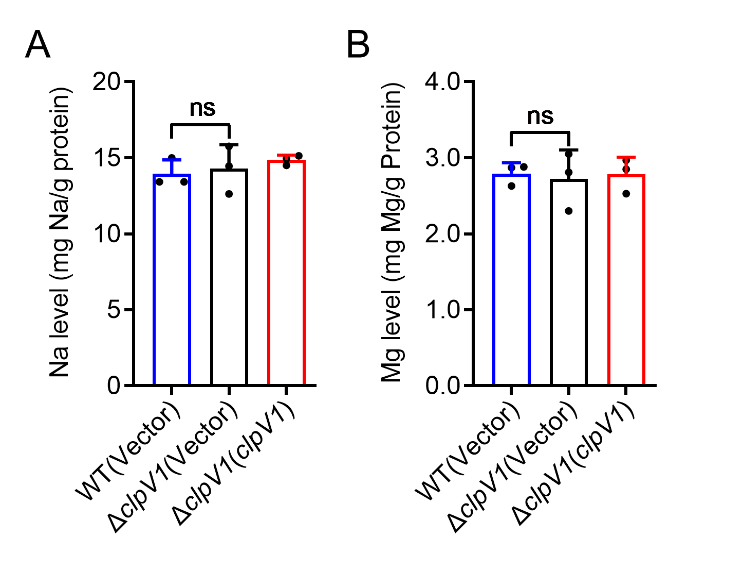


# Figure S2 T6SS1 was not involved in Na^+^ and Mg^2+^ accumulation in *C*. *pinatubonensis* under oxidative stress conditions. A and B, Stationary phase *C*. *pinatubonensis* strains were exposed to 0.5 mM H_2_O_2_ for 20 min in M9 medium, and Na^+^ (A) and Mg^2+^ (B) associated with bacterial cells were measured by ICP-MS. Data represent the mean ± SD of three biological replicates, each of which was performed in three technical replicates. ns, not significant.


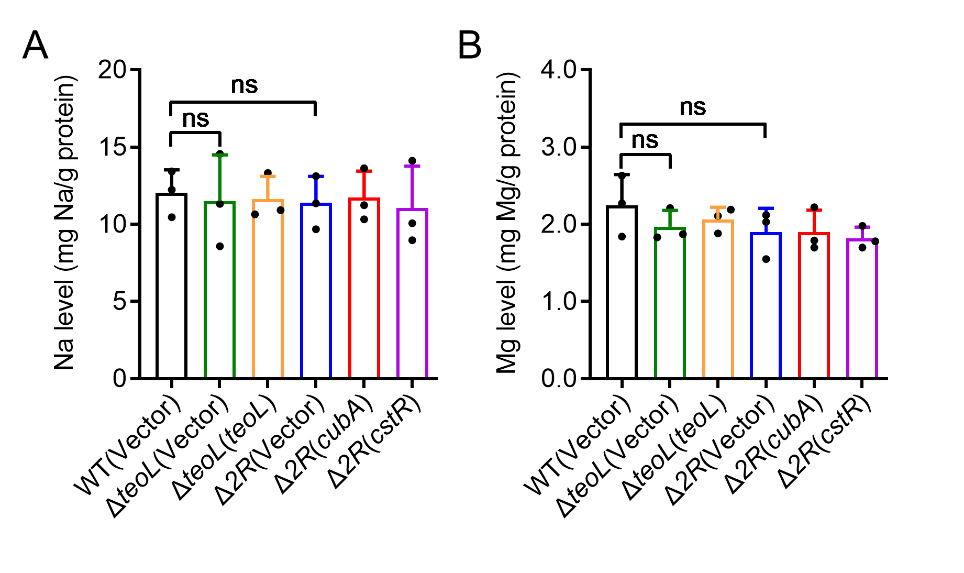


# Figure S3 TeoL, CubR, and CstR were not involved in Na^+^ and Mg^2+^ accumulation in *C*. *pinatubonensis* under oxidative stress conditions. A and B, Stationary phase *C*. *pinatubonensis* strains were exposed to 0.5 mM H_2_O_2_ for 20 min in M9 medium, and Na^+^ (A) and Mg^2+^ (B) associated with bacterial cells were measured by ICP-MS. Data represent the mean ± SD of three biological replicates, each of which was performed in three technical replicates. ns, not significant.


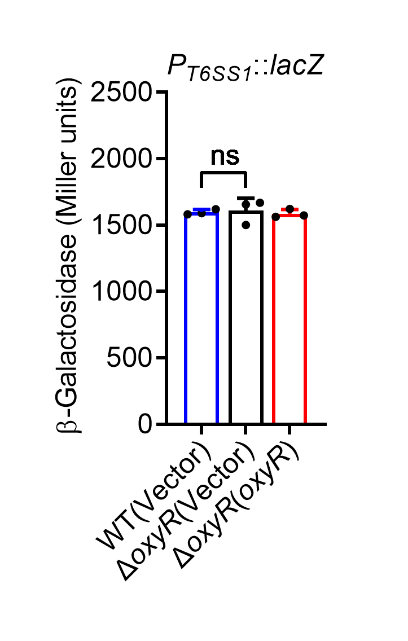


# Figure S4 OxyR does not affect the *cub* expression. β-galactosidase activity of *cub* promoter from chromosomal *lacZ* fusions in relevant *C*. *pinatubonensis* strains grown to stationary phase in M9 medium were measured. Data represent the mean ± SD of three biological replicates, each of which was performed with three technical replicates. ns, not significant.


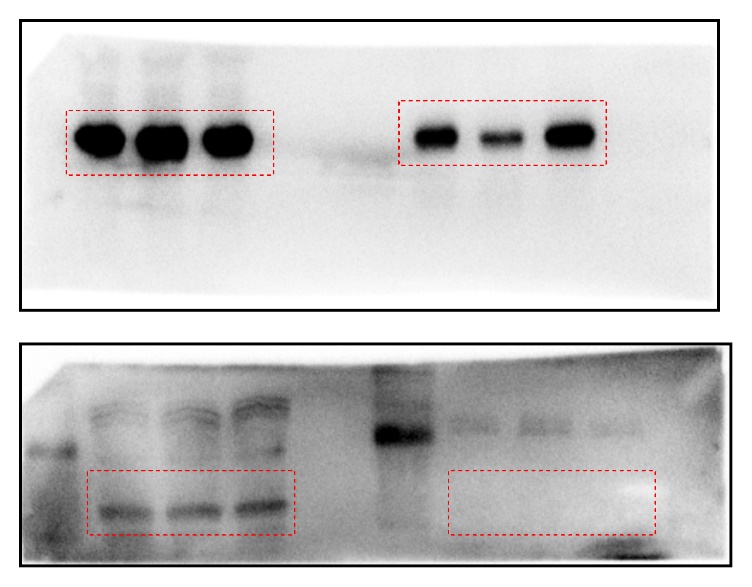


**Figure S5 Uncropped versions of Figure 2F.**

**Table S1 Bacterial strains and plasmids.**

| **Strain or plasmid** | **Relevant characteristics** | **Reference** |
| --- | --- | --- |
| ***E. coli*** |  |  |
| S17-1 *λ pir* | *λ*-pir lysogen of S17-1, *thi pro hsdR hsdM^+^ recA* RP4-Tc::Mu-Km::Tn7 | ([1](#_ENREF_1)) |
| BL21(DE3) | Host for expression vector pET28a | Novagen |
| DH5α | FΦ80Δ*lacZ*ΔM15/Δ(*lacZYA-argF*)*U169recA1 endA1 hsdR17* | Lab stock |
| ***C. pinatubonensis*** |  |  |
| *C. pinatubonensis* | Wild-type *Cupriavidus pinatubonensis* JMP134 | This study |
| Δ*oxyR* | *oxyR* gene deleted in *C*. *pinatubonensis* | This study |
| Δ*clpV1* | *clpV1* gene deleted in *C*. *pinatubonensis* | ([2](#_ENREF_2)) |
| Δ*katG* | *katG* gene deleted in *C*. *pinatubonensis* | This study |
| Δ*sod2* | *sod2* gene deleted in *C*. *pinatubonensis* | This study |
| Δ*teoL* | *teoL* gene deleted in *C*. *pinatubonensis* | ([2](#_ENREF_2)) |
| Δ*feoB* | *feoB* gene deleted in *C*. *pinatubonensis* | ([2](#_ENREF_2)) |
| Δ*cubA*Δ*cstR* (Δ*2R*) | *cubA/cstR* genes deleted in *C*. *pinatubonensis* | ([2](#_ENREF_2)) |
| Δ*teoL*Δ*2R* | *teoL*/*cubA*/*cstR* genes deleted in *C*. *pinatubonensis* | This study |
| Δ*2Fe*Δ*clpV1* | *cubE/feoB/clpV1* genes deleted in *C*. *pinatubonensis* | ([2](#_ENREF_2)) |
| WT(P*_T6SS1_*::*lacZ*) | Wild-type *C*. *pinatubonensis* containing pK18-P*_T6SS1_*::*lacZ*, Km^r^ | ([2](#_ENREF_2)) |
| WT(Vector) | Wild-type *C*. *pinatubonensis* containing pBBR1MCS-2/5, Km^r^/Gm^r^ | ([3](#_ENREF_3)) |
| Δ*oxyR*(Vector) | Δ*oxyR* containing pBBR1MCS-5, Gm^r^ | This study |
| Δ*oxyR*(*oxyR*) | Δ*oxyR* containing pBBR1MCS-5-*oxyR*, Gm^r^ | This study |
| Δ*oxyR*(*oxyR*^C199A, C208A^) | Δ*oxyR* containing pBBR1MCS-5-*oxyR*^C199A, C208A^, Gm^r^ | This study |
| WT(Vector, *hcp1*-*vsvg*) | Wild-type *C*. *pinatubonensis* containing pBBR1MCS-5 and pME6032-*hcp1*-*vsvg*, Km^r^/Gm^r^, Tc^r^ | This study |
| Δ*oxyR*(Vector, *hcp1*-*vsvg*) | Δ*oxyR* containing pBBR1MCS-5 and pME6032-*hcp1*-*vsvg*, Gm^r^, Tc^r^ | This study |
| Δ*oxyR*(*oxyR*, *hcp1*-*vsvg*) | Δ*oxyR* containing pBBR1MCS-5-*oxyR* and pME6032-*hcp1*-*vsvg*, Gm^r^, Tc^r^ | This study |
| Δ*clpV1*(Vector) | Δ*clpV1* containing pBBR1MCS-2, Km^r^ | ([2](#_ENREF_2)) |
| Δ*clpV1*(*clpV1*) | Δ*clpV1* containing pBBR1MCS-2-*clpV1*, Km^r^ | ([2](#_ENREF_2)) |
| Δ*katG*(Vector) | Δ*katG* containing pBBR1MCS-2, Km^r^ | This study |
| Δ*katG*(*katG*) | Δ*katG* containing pBBR1MCS-2-*katG*, Km^r^ | This study |
| Δ*sod2*(Vector) | Δ*sod2* containing pBBR1MCS-2, Km^r^ | This study |
| Δ*sod2*(*sod2*) | Δ*sod2* containing pBBR1MCS-2-*sod2*, Km^r^ | This study |
| Δ*teoL*(Vector) | Δ*teoL* containing pBBR1MCS-2, Km^r^ | ([2](#_ENREF_2)) |
| Δ*teoL*(*teoL*) | Δ*teoL* containing pBBR1MCS-2-*teoL*, Km^r^ | ([2](#_ENREF_2)) |
| Δ*feoB*(Vector) | Δ*feoB* containing pBBR1MCS-2, Km^r^ | This study |
| Δ*2R*(Vector) | Δ*2R* containing pBBR1MCS-2, Km^r^ | ([2](#_ENREF_2)) |
| Δ*2R*(*cubA*) | Δ*2R* containing pBBR1MCS-2-*cubA*, Km^r^ | ([2](#_ENREF_2)) |
| Δ*2R*(*cstR*) | Δ*2R* containing pBBR1MCS-2-*cstR*, Km^r^ | ([2](#_ENREF_2)) |
| Δ*teoL*Δ*2R*(Vector) | Δ*teoL*Δ*2R* containing pBBR1MCS-2, Km^r^ | This study |
| Δ*teoL*Δ*2R*(*teoL*) | Δ*teoL*Δ*2R* containing pBBR1MCS-2-*teoL*, Km^r^ | This study |
| Δ*teoL*Δ*2R*(*cubA*) | Δ*teoL*Δ*2R* containing pBBR1MCS-2-*cubA*, Km^r^ | This study |
| Δ*teoL*Δ*2R*(*cstR*) | Δ*teoL*Δ*2R* containing pBBR1MCS-2-*cstR*, Km^r^ | This study |
| Δ*2Fe*Δ*clpV1*(Vector) | Δ*2Fe*Δ*clpV1* containing pBBR1MCS-2, Km^r^ | This study |
| Δ*2Fe*Δ*clpV1*(*clpV1*) | Δ*2Fe*Δ*clpV1* containing pBBR1MCS-2-*clpV1*, Km^r^ | This study |
| Δ*2Fe*Δ*clpV1*(*cubE*) | Δ*2Fe*Δ*clpV1* containing pBBR1MCS-2-*cubE*, Km^r^ | This study |
| **Plasmid** |  |  |
| pK18*mobsacB* | *sacB*-based gene replacement vector, Km^r^ | ([4](#_ENREF_4)) |
| pK18-Δ*oxyR* | Construct used for in-frame deletion of *oxyR*, Km^r^ | This study |
| pK18-Δ*clpV1* | Construct used for in-frame deletion of *clpV1*, Km^r^ | ([2](#_ENREF_2)) |
| pK18-Δ*katG* | Construct used for in-frame deletion of *katG*, Km^r^ | This study |
| pK18-Δ*sod2* | Construct used for in-frame deletion of *sod2*, Km^r^ | This study |
| pK18-*P_T6SS1_*::*lacZ* | For *T6SS1* promoter fusion to *C*. *pinatubonensis*, Km^r^ | ([2](#_ENREF_2)) |
| pBBR1MCS-2 | Broad-host-range vector, Km^r^ | ([5](#_ENREF_5)) |
| pBBR1MCS-2-*clpV1* | *clpV1* cloned into pBBR1MCS-2 for complementation, Km^r^ | ([2](#_ENREF_2)) |
| pBBR1MCS-2-*katG* | *katG* cloned into pBBR1MCS-2 for complementation, Km^r^ | This study |
| pBBR1MCS-2-*sod2* | *sod2* cloned into pBBR1MCS-2 for complementation, Km^r^ | This study |
| pBBR1MCS-2-*teoL* | *teoL* cloned into pBBR1MCS-2 for complementation, Km^r^ | ([2](#_ENREF_2)) |
| pBBR1MCS-2-*cubA* | *cubA* cloned into pBBR1MCS-2 for complementation, Km^r^ | ([3](#_ENREF_3)) |
| pBBR1MCS-2-*cstR* | *cstR* cloned into pBBR1MCS-2 for complementation, Km^r^ | ([2](#_ENREF_2)) |
| pBBR1MCS-5 | Broad-host-range vector, Gm^r^ | ([5](#_ENREF_5)) |
| pBBR1MCS-5-*oxyR* | *oxyR* cloned into pBBR1MCS-5 for complementation, Gm^r^ | This study |
| pBBR1MCS-5-*oxyR*^C199A, C208A^ | *oxyR*^C199A, C208A^ cloned into pBBR1MCS-5 for complementation, Gm^r^ | This study |
| pME6032 | Shuttle vector, Tcr | ([6](#_ENREF_6)) |
| pME6032-*hcp1-vsvg* | pME6032 carrying *hcp1-vsvg* coding region, Tc^r^ | This study |
| pET28a | Expression vector with N-terminal His_6_ affinity tag, Km^r^ | Novagen |
| pET28a-*oxyR* | pET28a carrying *oxyR* coding region, Km^r^ | This study |

*Gm^r^, Km^r^ and Tc^r^ represent resistance to gentamycin kanamycin and tetracycline at 10, 50 and 20 μg mL^-1^, respectively.

**Table S2 Primers used in this study**

| **Primers** | **5’-3’ sequence** | **Function** |
| --- | --- | --- |
| *oxyR*-1F-EcoRI | CGGAATTCTCGAGGAAAGCGAAGCCCTGCAGC | To generate  pK18-Δ*oxyR* |
| *oxyR*-1R | CGCGCCACGGCGACGATGTACTT |  |
| *oxyR*-2F | **AAGTACATCGTCGCCGTGGCGCG**CGGGGGTCAGGAAGTTGAATGCCA |  |
| *oxyR*-2R-SalI | ACGCGTCGACCGAGAGGAATCGGAGGAGCACGCG |  |
| *katG*-1F-BamHI | CGGGATCCGAGCAGCTTGCCGATGCCCAG | To generate  pK18-Δ*katG* |
| *katG*-1R | TTCGCTTCAGTCGACATGTGTTCCC |  |
| *katG*-2F | **GGGAACACATGTCGACTGAAGCGAA**GGCGGCCAACCGATAAGCGTC |  |
| *katG*-2R-EcoRI | CCGGAATTCATCCAGCTCCCAGCCAACGTCG |  |
| *sod2*-1F-BamHI | CGGGATCCCACGAAACGGACTTCACCATTGCAG | To generate  pK18-Δ*sod2* |
| *sod2*-1R | GGGGGAGCTTGTGTTCCATTGTTCTG |  |
| *sod2*-2F | **CAGAACAATGGAACACAAGCTCCCCC**TCGCAGGCTGATTCGTTTCAGCG |  |
| *sod2*-2R-EcoRI | CCGGAATTCCTATCCCGCAGCCAGCAGCAAA |  |
| *oxyR*-F-KpnI | CGGGGTACCATGACGCTCACCGAACTGAAGTACA | To generate  pBBR1MCS-5-*oxyR* |
| *oxyR*-R-SacI | CGAGCTCTCAAGCCGCCTCAGCCAACT |  |
| *oxyRM*-1F-EcoRI | GACGGTATCGATAAGCTTGATATCGAATTCATGACGCTCACCGAACTGAAGTACATCG | To generate  pBBR1MCS-5-*oxyR*^C199A, C208A^ |
| *oxyRM*-1R | ATGATCACGGAAGGCATGCCCGCT |  |
| *oxyRM*-2F | **AGCGGGCATGCCTTCCGTGAT**CATGTGCTTGGCGTTGCCCCCGAG |  |
| *oxyRM*-2R-BamHI | GGTGGCGGCCGCTCTAGAACTAGTGGATCCTCAAGCCGCCTCAGCCAACTCC |  |
| *oxyR*-F-EcoRI | CGGAATTCATGACGCTCACCGAACTGAAGTACA | To generate  pET28a-*oxyR* |
| *oxyR*-R-SalI | ACGCGTCGACTCAAGCCGCCTCAGCCAACT |  |
| *katG*-F-XhoI | CCGCTCGAGATGTCGACTGAAGCGAAGTGTCCGTT | To generate  pBBR1MCS-2-*katG* |
| *katG*-R-BglII | GAAGATCTTTATCGGTTGGCCGCCTCGAAG |  |
| *sod2*-F-XhoI | CCGCTCGAGATGGAACACAAGCTCCCCCCGC | To generate  pBBR1MCS-2-*sod2* |
| *sod2*-R-BamHI | CGGGATCCTCAGCCTGCGAAGTTCTGAGCGG |  |
| *hcp1-vsvg*-F-EcoRI | CGGAATTCATGGACACCATCATCCTCGAGATCACTG | To generate  pME6032-*hcp1-vsvg* |
| *hcp1-vsvg*-R-BglII | GAAGATCTTCATTTTCCTAATCTATTCATTTCAATATCTGTATAAGATCTGGAAACGGCCTTGTTGGTAGCCAG |  |
| *T6SS1-*EMSA*-*F | AAATGCATCGCTCTCAGA | EMSA |
| *T6SS1-*EMSA*-*R | AATCTTTGTGTTTCTGTTG |  |
| *T6SS1M-*EMSA*-*F | AAATGCATCGCTCTCAGAATACCCAAAGTGGCAGCGAAGGGCGCCATCGACTTTCG |  |
| *Q16S-*F | GGGGAGTACGGTCGCAAGA | qRT-PCR |
| *Q16S-*R | ATGTCAAGGGTAGGTAAGGTTT |  |
| *QtssM1*-F | AGGGCATCTCGTGGCACTTCT |  |
| *QtssM1-*R | AACTCGTCCCAGGGCTTTTCAT |  |
| *QvgrG1*-F | TGACGAGACCCGCACCAA |  |
| *QvgrG1*-R | TGACCAGATCGCCAGACACC |  |
| *QclpV1*-F | GTGCTGCTCGACGAAATGGA |  |
| *QclpV1*-R | GGATGACCGTGTTGCGGAAGT |  |
| *Qhcp1*-F | CCGCTGATGACCTACACGC |  |
| *Qhcp1*-R | CTCGGTGAAATCCAGACAGAACG |  |

Underlined sites indicate restriction enzyme cutting sites added for cloning. Letters in boldface denote the annealing regions for overlap PCR.

**Table S3 OxyR ortholog in different species**

| **Species** | **Protein ID** |
| --- | --- |
| *Cupriavidus pinatubonensis* | AAZ62166 |
| *Cupriavidus necator* | CAJ94185 |
| *Burkholderia thailandensis* | ABC36444 |
| *Collimonas fungivorans* | AEK60594 |
| *Rubrivivax gelatinosus* | BAL93433 |
| *Thiomonas arsenitoxydans* | CAZ87568 |
| *Achromobacter xylosoxidans* | ADP16648 |
| *Bordetella hinzii* | AKQ55557 |
| *Advenella mimigardefordensis* | AHG63982 |
| *Sutterella megalosphaeroides* | BBF24010 |
| *Pseudomonas aeruginosa* | NP_254031 |
| *Halomonas beimenensis* | ATJ84728 |
| *Thalassolituus oleivorans* | CCU70619 |
| *Marinobacter adhaerens* | ADP96072 |
| *Nitrospira defluvii* | CBK39843 |
| *Azoarcus olearius* | ANQ83187 |
| *Sulfuritalea hydrogenivorans* | BAO27924 |
| *Chromobacterium violaceum* | AAQ61042 |
| *Laribacter hongkongensis* | ACO75513 |
| *Ephemeroptericola cinctiostellae* | AXF84344 |
| *Escherichia coli* | NP_418396 |
| *Shigella flexneri* | NP_709765 |
| *Salmonella enterica* | NP_463004 |
| *Pectobacterium atrosepticum* | AIK16002 |
| *Serratia plymuthica* | AEF47962 |
| *Xenorhabdus bovienii* | CBJ83369 |
| *Vibrio parahaemolyticus* | BAC61015 |
| *Grimontia hollisae* | AMG30402 |
| *Thermomonas carbonis* | QNN68851 |
| *Luteibacter rhizovicinus* | APG03008 |
| *Hydrogenovibrio marinus* | BBN58457 |

**References**

1. Simon R, Priefer U, Pühler A. 1983. A broad host range mobilization system for in vivo genetic engineering: transposon mutagenesis in gram negative bacteria. Bio/technology 1:784.

2. Li C, Zhu L, Wang D, Wei Z, Hao X, Wang Z, Li T, Zhang L, Lu Z, Long M, Wang Y, Wei G, Shen X. 2022. T6SS secretes an LPS-binding effector to recruit OMVs for exploitative competition and horizontal gene transfer. ISME J 16:500-510.

3. Li C, Zhu L, Pan D, Li S, Xiao H, Zhang Z, Shen X, Wang Y, Long M. 2019. Siderophore-mediated iron acquisition enhances resistance to oxidative and aromatic compound stress in *Cupriavidus necator* JMP134. Appl Environ Microbiol 85:e01938-18.

4. Schafer A, Tauch A, Jager W, Kalinowski J, Thierbach G, Puhler A. 1994. Small mobilizable multi-purpose cloning vectors derived from the *Escherichia coli* plasmids pK18 and pK19: selection of defined deletions in the chromosome of *Corynebacterium glutamicum*. Gene 145:69-73.

5. Kovach ME, Elzer PH, Hill DS, Robertson GT, Farris MA, Roop RM, 2nd, Peterson KM. 1995. Four new derivatives of the broad-host-range cloning vector pBBR1MCS, carrying different antibiotic-resistance cassettes. Gene 166:175-6.

6. Heeb S, Blumer C, Haas D. 2002. Regulatory RNA as mediator in GacA/RsmA-dependent global control of exoproduct formation in *Pseudomonas fluorescens* CHA0. J Bacteriol 184:1046-56.
